# The argument for integrating vector control with multiple drug administration campaigns to ensure elimination of lymphatic filariasis

**DOI:** 10.1186/1475-2883-5-10

**Published:** 2006-08-16

**Authors:** TR Burkot, DN Durrheim, WD Melrose, R Speare, K Ichimori

**Affiliations:** 1 Centers for Disease Prevention and Control, Division of Parasitic Diseases, 4770 Buford Highway, Mailstop F42, Atlanta, Georgia 03041, USA; 2 WHO Lymphatic Filariasis Collaborating Center, James Cook University, Townsville, QLD, 4811, Australia; 3 Hunter New England Population Health, Locked Bag 10, Wallsend New South Wales 2287, Australia; 4 PacELF, World Health Organization, PO Box 113, Suva, Fiji Islands

## Abstract

**Background:**

There is a danger that mass drug administration campaigns may fail to maintain adequate treatment coverage to achieve lymphatic filariasis elimination. Hence, additional measures to suppress transmission might be needed to ensure the success of the Global Program for the Elimination of Lymphatic Filariasis.

**Discussion:**

Vector control successfully eliminated lymphatic filariasis when implemented alone or with mass drug administration. Challenges to lymphatic filariasis elimination include uncertainty of the exact level and duration of microfilarial suppression required for elimination, the mobility of infected individuals, consistent non-participation of some infected individuals with mass drug administration, the possible development of anti-filarial drug resistance and treatment strategies in areas co-endemic with loasis.

Integration of vector control with mass drug administration can address some of these challenges. The potential benefits of vector control would include: (1) the ability to suppress filariasis transmission without the need to identify all individual 'foci of infection'; (2) minimizing the risk of reestablishment of transmission from imported microfilaria positive individuals; and (3) decreasing the risk of dengue or malaria transmission where, respectively, *Aedes *or *Anopheles *are lymphatic filariasis vectors.

**Summary:**

With adequate sustained treatment coverage, mass drug administration should meet the criteria for elimination of lymphatic filariasis. However, it may be difficult to sustain sufficiently high mass drug administration coverage to achieve lymphatic filariasis elimination in some areas, particularly, where *Aedes *species are the vectors. Since vector control was effective in controlling and even eliminating lymphatic filariasis transmission, integration of vector control with mass drug administration will ensure the sustainability of transmission suppression and thereby better ensure the success of national filariasis elimination programs. Although trials of some vector control interventions are needed, proven vector control strategies are ready for immediate integration with mass drug administration for many important vectors. Vector control is the only presently available additional lymphatic filariasis control measure with the potential for immediate implementation.

## Background

Efforts to control lymphatic filariasis (LF) have a long history in the Pacific island countries and territories. Although only *Wuchereria bancrofti *is found in the South Pacific, the epidemiology of transmission is complex. In Micronesia and in the Melanesian countries of Papua New Guinea (PNG) and Vanuatu, the parasite is periodic. Transmission is by *Culex quinquefasciatus *in Micronesia, while the members of the *Anopheles punctulatus *complex transmit both malaria and *Wuchereria bancrofti *in PNG and Vanuatu. In New Caledonia, *W. bancrofti *is aperiodic and transmitted by the night and day-time biting *Aedes vigilax*. Where *W. bancrofti *is subperiodic, it may be transmitted by various *Aedes *vectors (Table 1). From Fiji to French Polynesia, 15 *Aedes *vectors are reported (six vectors are found in Fiji alone). Except for *Ae. polynesiensis *and *Ae. vigilax*, little is known about the ecology of these mosquitoes. *Ae. polynesiensis *is arguably the most important LF vector in the Pacific, in part because it exhibits a characteristic known as 'limitation', whereby the percentage of microfilaria (mf) which develop to stage 3 larvae increases with decreasing densities of mf [1]. For this reason, *Ae polynesiensis *may pose the greatest challenge to LF elimination in the region.

**Table 1:** Reported *Aedes *vectors of lymphatic filariasis in the Pacific

Vector	Countries where found
*Aedes cooki*	Niue
*Aedes fijiensis*	Fiji
*Aedes horrensces*	Fiji
*Aedes kochi*	Papua New Guinea
*Aedes marshallensis*	Kiribati
*Aedes oceanicus*	Tonga, Samoa
*Aedes polynesiensis*	American Samoa, Samoa, Cook Islands, Tokelau, Tuvalu, French Polynesia, Wallis and Futuna, Fiji
*Aedes pseudoscutellaris*	Fiji
*Aedes rotumae*	Rotuma Island in Fiji
*Aedes samoanus*	Samoa
*Aedes tabu*	Tonga
*Aedes tongae*	Tonga
*Aedes tutuilae*	Samoa
*Aedes upolenis*	Samoa
*Aedes vigilax*	New Caledonia, Fiji

The Pacific Programme for the Elimination of Lymphatic Filariasis (PacELF) was the first regional LF elimination programme established [2]. Following the Global Lymphatic Filariasis Elimination Programme recommendations for stopping transmission, PacELF has targeted >80% of populations in endemic areas with diethylcarbamazine (DEC) and albendazole annually for at least five years. Evidence that the DEC/albendazole combination will be more successful than monotherapy with DEC against adult worms is supported by reductions in both adult worm antigen levels and in clinical reactions in infected humans receiving the drug combination [3]. This combination therapy appears to be more effective in reducing the prevalence and density of mf for longer time periods than DEC alone [4].

Under PacELF, national MDAs were undertaken in the following countries where *Aedes *species are important vectors of *W. bancrofti*: American Samoa, Cook Islands, Fiji, French Polynesia, Niue, Samoa, Tonga, Tuvalu, and Wallis and Futuna (Tokelau is no longer considered to be endemic). By the end of 2005, five or more rounds of MDA had been completed in the Cook Islands, French Polynesia, Niue, Samoa and Tonga. Samoa is the only country to have completed its prevalence assessment after five rounds of MDA, with annual coverage ranging from 57% to 90%. In addition to these programmes where *Aedes *transmit filaria, MDA has been undertaken in countries where *Anopheles *(Vanuatu and Papua New Guinea) and *Cx quinquefasciatus *(Federated States of Micronesia, Kiribati, Marshall Islands and Palau) are the primary vectors.

**Figure 1:**
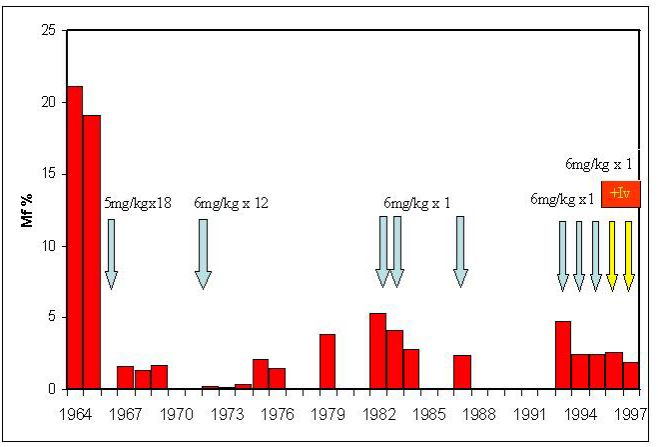
**Impact of DEC on microfilaria prevalence rates in Samoa: 1964–1994**. DEC-based MDAs were administered at either 5 or 6 mg/kg body weight as either a single annual dose or monthly for 12 or 18 months, as indicated. Ivermectin (+Iv) was administered in 1996 and 1997 at a dose of 200 μg/kg body weight with a single dose of DEC at 6 mg/kg body weight [5].

Previous MDA based campaigns using only DEC in Samoa and French Polynesia suggest that caution should be exercised on deciding when to stop MDA. These campaigns often succeeded in achieving significant reductions in mf rates and intensities and LF associated morbidity [5-7]. In Samoa, five extensive campaigns using DEC, including 12–18 month treatments in 1966 and 1971, and single annual doses in 1982, 1983 and 1986, reduced the mf rate from 21% in 1964 to 2.3% in 1987 (Figure 1). Mf rates declined to 0.14% in 1974, following the second DEC campaign, but rebounded to 2.1% within two years [5].

In French Polynesia, twice yearly DEC chemotherapy (6 mg/kg) was administered for 34 years beginning in 1955 to an average of 85% of the population on Maupiti island (excluding the years 1960–67 and 1970–74) [6]. In addition, mosquito control using DDT (1955–1957) and larval source reduction (1955–1970) was implemented. Despite these efforts, a comprehensive survey in 2000 found that 0.4% of residents still had circulating mf and 4.6% had antigenaemia [6].

After cessation of the MDA campaigns in Samoa and French Polynesia, mf rates increased [5,8]. While these extensive campaigns succeeded in reducing filariasis transmission, elimination of the parasite was not achieved. Mf resurgence was attributed to poor compliance, inadequate campaign durations and failure of DEC to completely kill or sterilize adult *W. bancrofti *in treated people (Chow CY: Filariasis vectors and their control in the South Pacific. In *4th Joint WHO/SPC seminar on Filariasis and Vector Control*. Apia, W. Samoa, WPR/Fil/9. Manila, World Health Organization, 1974). A subsequent analysis of LF positive individuals on Maupiti suggested that continued transmission can be attributed to individuals who persistently failed to participate in the MDA programme (Nguyen, personal communication).

The historic MDA campaigns in Samoa and French Polynesia, described above, relied on DEC alone. The present GPELF (outside Africa) and PacELF strategies rely on the treatment combination of DEC with albendazole that is reportedly more effective against *W. bancrofti*. However, recent systematic reviews question whether there really is an improved effectiveness of DEC and albendazole compared to DEC alone. These analyses suggest that the addition of albendazole to DEC does not significantly improve the microfilaricidal [9,10] or macrofilariacidal activity of DEC [9]. These disconcerting conclusions were based on a limited number of studies. A more recent study found significantly greater reductions in microfilaemia intensity and antigenaemia prevalence in persons treated with DEC and albendazole compared to DEC alone [11]. Additional studies are clearly needed. There can be little doubt that administration of albendazole with DEC benefits the people receiving the drugs. However, concerns remain about the ability of MDA alone to succeed in eliminating LF without ongoing universal coverage of the eligible target populations, particularly in areas where efficient vectors such as *Ae. polynesiensis *exist. Hence, there is a need to consider complementary control strategies to ensure the success of the global LF elimination campaign. The only presently available alternative to filaricidal treatments (MDA-based campaigns or distribution of DEC-medicated salt) is vector control.

Vector control was the primary tool for controlling filariasis in the Pacific before effective antifilarial drugs were available and even after effective antifilarials became available vector control was preferred by Pacific island ministries and departments of health because MDA campaigns were considered too labour intensive [12].

Elimination of filariasis using vector control alone has already been successfully documented in the Pacific. Where *Anopheles *species are the vectors of malaria and filariasis, filariasis was eliminated from areas where indoor residual spraying (IRS) with DDT to control malaria was undertaken in areas of Papua New Guinea [13] and throughout the Solomon Islands [14,15].

*W. bancrofti *was also eliminated from Australia by sanitation campaigns that controlled the major vector, *Cx quinquefasciatus *[16]. Vector control also played a significant role in elimination of LF from Japan [17]. Demonstration of the impact of polystyrene beads on *Cx quinquefasciatus *populations and hence on LF transmission in Zanzibar and India where pit latrines or soakage pits are major breeding sites suggests that control of *Cx quinquefasciatus *can augment MDA to suppress transmission [18-20].

*Aedes polynesiensis *has been successfully controlled in a small number of trials of limited scope in the Pacific (Table 2). *Mesocyclops aspericornis *reduced the number of *Ae. polynesiensis *larvae in treated crab holes by 98% [21]. The effectiveness of larval source-reduction campaigns against *Ae. polynesiensis *has been repeatedly demonstrated in French Polynesia [22] (Kessel JF: *Combined control methods in filariasis*. Manila, World Hlth Org, unpublished report, 1965 (FIL/WP/16.65), particularly when integrated with other measures [23] including removal of vegetation to facilitate discovery of breeding sites (Byrd EE, St Amant LS: Studies on the epidemiology of filariasis on Central and South Pacific Islands. In *SPC Technical Paper*, 1959, 125:52–55) and MDA campaigns [24]. Such campaigns can be effective even though they are labour intensive (Hairston N: *Assessment of filariasis in Western Samoa*. Assignment report. Manila, World Hlth Org 1973) and despite the rapidity with which breeding sites reappear after campaigns are concluded [25].

**Table 2:** Summary of field trials for controlling Aedes in the Pacific

Vector*	Breeding site	Country	Control method	% Reduction in Mosquitoes	Reference
*Ae. polynesiensis*	Crab holes	Fiji	1% Lindane and plugging of crab holes	0% on biting	26
*Ae. polynesiensis*	All types	French Polynesia	Breeding site elimination within 100 yd. of village	80% to 90%	Kessel JF, 1965, unpublished
*Ae. polynesiensis*	All types	French Polynesia	Breeding site elimination; vegetation control	81% of biting Adults	Byrd EE, St Amant LS, 1959, unpublished
*Ae. polynesiensis *and *Ae. samoanus*	All types	Samoa	DDT spray of breeding site and fogging of houses/bush	64% of biting adults	Suzuki, Stone, 1976, unpublished
*Ae. polynesiensis *and *Ae. samoanus*	Not reported	Samoa	Abate larvicide and malathion fog	60% of biting adults	Chow CY, 1974, unpublished
*Ae. polynesiensis*	Not reported	American Samoa	DDT house spraying; aerial spraying every 14 days	0% of adults after 14 days	Wharton JD, Jachowski LA, 1980, unpublished
*Ae. aegypti *and *Ae. polynesiensis*	Cisterns, wells, drums	French Polynesia	Integrated control (Abate, sealing drums, polystyrene beads, *Poecillia reticulata*)	84% of adults	23
*Ae. polynesiensis*	Crab holes	French Polynesia	*Mesocyclops aspericornis*	98% of larvae	21
*Ae. polynesiensis*	Crab holes	French Polynesia	Insecticide impregnated crab bait	86% of larvae	39

However, not all vector control interventions are successful. Insecticide fogging campaigns had minimal impacts on *Ae. polynesiensis *biting rates, with reductions of less than 64% reported in three unpublished trials (Suzuki T, Stone F: *Laboratory and field tests of insecticides against vector mosquitos of subperiodic filariasis in Western Samoa*. Unpublished report to WHO, 1976; Chow CY: Filariasis vectors and their control in the South Pacific. In *4th Joint WHO/SPC seminar on Filariasis and Vector Control*. Apia, W. Samoa, WPR/Fil/9. Manila, World Health Organization, 1974; Wharton JD, Jachowski LA. In: Zahar, King and Chow. *A review and annotated bibliography on subperiodic bancroftian filariasis with special reference to its vectors in Polynesia, South Pacific*. Manila, World Health Organization, 1980). There have not been any studies on controlling the salt marsh breeding *Ae. vigilax *in the Pacific islands. While runnels (ditches designed to allow tidal flushing of salt marshes) have decreased larval numbers in Australia [26], the impact of runnels on *Ae. vigilax *biting rates needs to be evaluated before widespread programmatic application can be considered.

## Discussion

Despite significant progress towards LF elimination, a number of challenges to MDA-based LF elimination programmes remain. Firstly, we do not know the exact level and duration of mf suppression required for elimination to be achieved. A target goal reduction of mf prevalence below 1% for transmission elimination may not be an appropriate target where *Aedes *mosquitoes are vectors. In Samoa, mf rates were less than 0.33% between 1972 and 1974, but LF prevalence rates rose soon after MDA was stopped. It is likely that foci of mf positive individuals capable of initiating resurgence of LF will remain after five or more MDAs with DEC and albendazole. Five years of MDA with DEC and albendazole in Egypt do not appear to have been sufficient to eliminate LF transmission in areas that had high baseline infection rates [27]. Effective tools for locating such foci in countries with a low overall residual mf prevalence after five or more annual MDAs have yet to be developed.

A second significant challenge in the Pacific is population mobility. Migration is particularly common in many of the Pacific islands; more Cook Islanders live in New Zealand than on the main island of Rarotonga. Samoans frequently travel between Samoa and American Samoa for economic opportunities and to visit relatives and friends living in the neighbouring country. A significant proportion of internal migrants in Papua New Guinea are mf positive [28]. Such migrants are likely to miss annual MDA treatments, thereby raising the possibility of reintroduction of LF.

A third challenge is the presence of individuals whose occupational or social behaviour places them at risk of infection or who consistently fail to participate in MDA, placing their communities at risk of ongoing transmission [29,30]. Merely increasing the number of years of MDA campaigns will not reach these individuals. A better understanding of their perceptions and priorities will allow tailoring of elimination messages and interventions so that they are locally appropriate and acceptable [31].

A fourth challenge is the potential for *W. bancrofti *to develop resistance to either DEC or albendazole. Albendazole resistance is already common amongst helminths of veterinary importance. Although there is currently no evidence of resistance to DEC or albendazole in areas where LF elimination programmes are under way, no reliable assay system is currently available to allow assessment of resistance in filarial nematodes. Resistance is more likely to appear in an MDA programme after several rounds of treatment when success appears to be in sight.

A fifth challenge is areas where LF and loiasis are co-endemic [30]. MDA cannot be implemented in these areas until mf densities are reduced to levels where significant adverse reactions from MDA will not occur. This is due to severe adverse events associated with treatment with Mectizan^® ^(ivermectin) in community directed treatment in onchocerciasis if patients have high microfilarial loads of *Loa loa*.

These challenges can be met by integrating vector control with MDA for LF elimination [32]. Vector control is the only presently available adjunct LF control measure with the potential for immediate implementation. Although treatment with doxycycline has been shown to eliminate mf and to have macrofilaridal activity, mass treatment is not practical due to the logistic difficulties of delivering daily doxycycline treatments of 200 mg for 6 to 8 weeks [33]. In addition, doxycycline is also contraindicated for use in children under 8 years and in pregnant women.

National scale vector control programmes would have multiple potential benefits for LF elimination programmes. These include (1) the ability to suppress LF transmission without the need to identify all individual 'foci of infection'; (2) minimizing the risk of reestablishment of transmission from imported mf positive individuals; and (3) reducing the spread of any DEC or albendazole resistant *W. bancrofti *which might emerge. Furthermore, control measures targeting vectors will also decrease the risk of dengue or malaria transmission where, respectively,*Aedes *or *Anopheles *are the LF vectors. The reduction of nuisance in addition to vector mosquito biting is likely to enhance community support for ELF programmes. Implementation of vector control strategies as adjuncts to the MDA campaigns will better ensure the success of the elimination efforts and enhance the prospects for sustainable benefit.

Mosquito surveillance and control as adjuncts to MDA are already included in many country filariasis elimination plans even though they may not be actively implemented. Among PacELF countries, activities commonly mentioned include larval surveys for filariasis vectors, environmental sanitation to reduce mosquito breeding sites, use of mosquito nets and ultra-low-volume (ULV) spraying against adult mosquitoes. We need to support vector control efforts to ensure that the limited resources available to ministries of health are spent effectively. There are a number of vector control strategies whose efficacy against transmission of LF has been demonstrated. These include the use of insecticide treated mosquito nets where *Anopheles *are LF vectors [34,35] and the use of polystyrene beads for control of *Culex *vectors that breed in pit latrines or soakage pits[18]. These strategies for those vectors are ready for widespread implementation. Mosquito nets should also suppress LF transmission whenever the vectors are night-biting (i.e., *Anopheles, Culex, Mansonia, Ae. vigilax*), but the degree of the impact needs to be confirmed in controlled trials before implementation on a country basis should be advocated.

Vector control methods for LF are more than just mosquito nets and polystyrene beads. Recommendations for research and control trials were presented at a "LF Research Forum" in 2003 [36]. Examples of priorities for vector control research included the evaluation of insecticide treated materials (including, but not limited to nets) on *Culex *and source reduction on *Aedes *vectors.

Dengue control campaigns are now primarily based on larval source reduction to limit transmission by the primary vector, *Ae. aegypti*. While important behavioural differences exist between *Ae. aegypti *and *Ae. polynesiensis*, there are sufficient similarities between the two species (both bite in the daytime and breed in containers) to allow LF programme managers to draw some lessons from the anti *Ae. aegypti *campaigns for *Ae. polynesiensis *control. Integration of LF with dengue control programmes in *Aedes *transmission areas makes sense. Similarly, malaria control programmes in *Anopheles *transmission areas would also suppress LF transmission though insecticide treated mosquito nets and/or IRS if LF elimination and malaria control programmes were integrated.

In addition, control of the night-biting but predominantly outdoor-feeding *Mansonia *vectors should include evaluations of the impact on the adult populations of vegetation removal from rivers and ponds (the larvae obtain air from plants via a modified siphon) as well as the use of fish to reduce the larval populations [37]. Similarly, studies to measure reductions of *Ae vigilax *adults by environmental modifications of salt marshes will be required before vector control can be advocated for extensive geographic areas.

Before implementing a vector control strategy on a country-wide basis, the effectiveness of interventions needs to be validated at the population level, so that limited resources are targeted for optimal control. It is noteworthy that China, the first country to apply for formal verification of interruption of transmission used an integrated control strategy that included vector control [38].

It has been argued that vector control is not cost-effective for LF elimination. However, the cost of failing to achieve final elimination of LF will far exceed the short-term costs of implementing vector control for LF elimination where it is needed.

## Summary

1. MDA alone should meet the criteria for elimination of LF in many areas, if adequate treatment coverage can be maintained. However, it may be difficult to sustain sufficiently high MDA coverage to achieve LF elimination where *Aedes *spp are the vectors or where *Culex *populations are abundant.

2. Vector control has been effective in controlling and even eliminating transmission of *W*. bancrofti either alone or when implemented in an integrated LF programme with MDA.

3. In addition to reducing the risk of the re-establishment of LF, *Aedes *and *Anopheles *control for LF will reduce the risk of dengue and malaria transmission, respectively.

4. Integration of LF with dengue and malaria control programmes where *Aedes *and *Anopheles *are the vectors, respectively, will enhance the sustainability and success of the LF elimination efforts.

5. Vector control is the only presently available adjunct LF control measure with the potential for immediate implementation.

6. There is still a need for larger scale trials of vector control interventions for some LF vectors.

7. However, there are vector control strategies that have proven effective in limiting the transmission of *W. bancrofti *that can be implemented immediately, including the use of insecticide treated mosquito nets and polystyrene beads for control of transmission by *Anopheles *and *Culex*, respectively.

## Competing interests

The author(s) declare that they have no competing interests.

## Authors' contributions

TRB conceived the main points presented in the manuscript and wrote the original manuscript draft. DND and KI provided unpublished evidence to support the main points presented in the manuscript as well as significant intellectual input into framing the major points presented. RS and WDM provided intellectual input into the arguments presented.
